# USP14 promotes colorectal cancer progression by targeting JNK for stabilization

**DOI:** 10.1038/s41419-023-05579-5

**Published:** 2023-01-24

**Authors:** Xue-Hua Du, Shao-Bo Ke, Xin-Yi Liang, Jie Gao, Xiao-Xiao Xie, Lin-Zhi Qi, Xue-Yi Liu, Guo-Yuan Xu, Xiao-Dong Zhang, Run-Lei Du, Shang-Ze Li

**Affiliations:** 1 School of Medicine, Chongqing University, Chongqing, 400030 China; 2 Hubei Key Laboratory of Cell Homeostasis, College of Life Sciences, Wuhan University, Wuhan, Hubei 430072 China; 3 Cancer Center, Renmin Hospital of Wuhan University, Wuhan, 430060 China

**Keywords:** Oncogenes, Cell signalling, Post-translational modifications

## Abstract

MAPK/JNK signaling is pivotal in carcinogenesis. However, ubiquitin-mediated homeostasis of JNK remains to be verified. Here, with results from RNA sequencing (RNA-seq) and luciferase reporter pathway identification, we show that USP14 orchestrates MAPK/JNK signaling and identify USP14 as a deubiquitinase that interacts and stabilizes JNK. USP14 is elevated in colorectal cancer patients and is positively associated with JNK protein and downstream gene expression. USP14 ablation reduces cancer cell proliferation in vitro and colorectal tumorigenesis in vivo by downregulating MAPK/JNK pathway activation. Moreover, USP14 expression is induced by TNF-α, forming a feedback loop with JNK and leading to tumor amplification. Our study suggests that elevated expression of USP14 promotes MAPK/JNK signaling by stabilizing JNK, which in turn augments colorectal carcinogenesis, indicating a potential therapeutic target for colorectal cancer patients with increased USP14 expression.

## Introduction

The mitogen-activated protein of the Ser/Thr kinase family (MAPK) is critical for transforming various extracellular signals into intracellular responses. The aberrant expression of MAPKs is closely related to development, cell fate determination, proliferation and survival and inflammation and therefore has been reported to be involved in many diseases ranging from all kinds of cancers to inflammatory diseases to nervous system diseases and metabolic diseases [1, 2]. c-Jun N-terminal kinase 1/2/3 (JNK1/2/3) belong to one of three classic MAPK subfamilies, and their activity is dependent on upstream MAPK kinases (MAPKKs) and MAPK kinase kinases (MAPKKKs) [3]. MKK4 and MKK7 are directly upstream of JNK, and they are activated in response to stress signals and inflammatory cytokines, respectively [4]. JNK is involved in the regulation of immune and inflammatory responses, cell growth, apoptosis, and tumor progression [5]. The AP-1 components c-jun/c-fos are major targets of JNK, which is phosphorylated, resulting in increased transcriptional activity to regulate the survival of cancer cells [6]. Many studies have reported that JNK is involved in colorectal cancer progression. The survival-promoting role of JNK is mainly mediated by crosstalk between JNK and other pathways, including the Wnt signaling pathway [7].

Aberrant expression and activation of JNK have been reported in various types of cancer [8]. Posttranscription modifications (PTMs) influence protein stability and conformation, as well as subsequent cellular sublocalization and enzyme catalytic activity and interplay [9]. The phosphorylation of JNK induces a conformational switch exposing the activated site and triggering a downstream signaling cascade [9, 10]. Ubiquitination is a PTM involved in a wide spectrum of signaling cascades, with ubiquitination of lysine 63 (K63) enhancing protein activity and ubiquitination of lysine 48 (K48) regulating protein stability [11, 12].

The removal of ubiquitin by deubiquitinating enzymes (DUBs) is essential in ubiquitin homeostasis to ensure the completion of various biological processes. Ubiquitin-specific peptidase 14 (USP14), a DUB family member, plays a critical role in synaptic development and neuromuscular junctions [13], innate immune defense [14–16], and hepatosteatosis [17]. In addition, USP14 has been reported to be involved in regulating cellular proliferation and apoptosis in epithelial ovarian cancer and oral squamous cell carcinoma [18, 19]. In prostate cancer, USP14 regulates cancer cell proliferation by deubiquitinating and stabilizing androgen receptors [20]. In lung tumorigenesis, USP14 regulates autophagy formation [21]. Although many reports have shown that highly expressed USP14 promotes cancer cell proliferation via the Wnt/β-catenin pathway and is associated with metastasis [22–24], the substrates and mechanism of USP14 as an oncogene still need to be explored.

In this study, through RNA sequencing (RNA-seq) and luciferase assays, we characterized the USP14 regulatory pathway and identified a new substrate of USP14. We revealed an oncogenic role for USP14 in colorectal cancer in vivo and in vitro: USP14 deubiquitinates and stabilizes JNK, which in turn promotes MAPK/JNK signaling cascade activation. In patients, the expression of USP14 was found to be upregulated and positively associated with JNK expression. Finally, we revealed a novel mechanism of USP14 in colorectal cancer development and progression.

## Materials and methods

### Study approval

All colorectal tissues from patients undergoing colorectal cancer (CRC) resection were collected immediately in a liquid nitrogen tank. Written informed consent was obtained from all subjects at the Tongji Medical College Huazhong University of Science & Technology in Wuhan. The animal experiments were approved by the Committee on Ethics in the Care and Use of Laboratory Animals of Wuhan University.

### Animal experiment model

Given the effect size and standard deviation, the method for determining the sample size in the animal studies was chosen in accordance with the Animal Research Committee’s suggestion. USP14-knockout mice often exhibit embryonic or early postnatal lethality. To obtain adult USP14-knockout mice, a refined strategy for conditional gene knockout that has been previously used and relies on the DNA recombinase Cre and its recognition (LoxP) sites. First, USP14^floxp/+^ mice (GemPharmatech, China) were obtained through the insertion of recombinase LoxP sites in intron 2 and intron 9 of USP14. Second, USP14^floxp/floxp^ mice were obtained by mating USP14^floxp/+^ mice. Next, to obtain intestinal-specific USP14-knockout mice (USP14^floxp/floxp^;Villin-Cre), USP14^floxp/floxp^ mice were crossed with intestinal-specific Cre transgenic mice (Villin-Cre) for at least two generations. The mice were genotyped by PCR analysis followed by sequencing. USP14^floxp/floxp^ and USP14^floxp/floxp^;Villin-Cre littermates were cohoused until weaning and then randomly separated into each groups for experiments. The investigators was blinded to the group allocation during the experiment. All mice (8–10 weeks old) were housed in a specific pathogen-free and temperature-controlled (23 ± 2 °C) environment with a 12-h light/dark cycle. Colorectal cancer in mice was induced by azoxymethane (AOM)/dextran sodium sulfate (DSS). USP14^floxp/floxp^ and USP14^floxp/floxp^;Villin-Cre mice (male, 8~10 weeks old, weighing 23–28 grams) were allocated to two groups, with 10 in each group. Briefly, mice were first injected intraperitoneally with 12 mg/kg AOM (Sigma–Aldrich, St. Louis, MO) dissolved in normal saline. Seven days after injection, the mice were given 2% DSS (35–50 kDa; MP Biochemicals, Solon, OH) in their drinking water for five consecutive days followed by regular drinking water for 7 days. Subsequently, the mice received a second injection of AOM (12 mg/kg). Seven days after the second AOM intervention, 2% DSS was again added to the drinking water for five consecutive days followed by regular water until day 81. During the model-development process, body weight, diarrhea, and hematochezia were monitored. The mice were sacrificed on day 81. All experimental procedures involving animals were performed in accordance with the Guide for the Care and Use of Laboratory Animals and were approved by the Committee on Ethics in the Care and Use of Laboratory Animals of Wuhan University and Animal Care and Use Committee of Wuhan University (approval number: AF016).

### Antibodies

The following primary antibodies were used in this study: anti-USP14 antibody (#11931, 1:1000), anti-JNK antibody (#9252, 1:1000), anti-C-Myc antibody (#18583, 1:1000), anti-Cyclin D2 antibody (#3741, 1:1000), anti-MDM2 antibody (#86934, 1:1000), and anti-C-Jun antibody (#9165, 1:1000), which were obtained from Cell Signaling Technology (Beverly, MA, USA) and anti-Flag antibody (M185-3L), anti-HA antibody (561), anti-c-MYC antibody (M192-3), anti-β-actin antibody (M177-3) and anti-GAPDH (M171-3), which were obtained from MBL (Nagoya, Japan).

### Plasmids

The plasmids pET28a-His-USP14, pHAGE-3×Flag-USP14, pHAGE-3×Flag-USP14C114A and pHAGE-3×HA-JNK2 were constructed according to the methods in the “Molecular Cloning Experiment Guide”. PCR primers are listed in the Supplemental Table 1.

### Cell culture and cell lines

HCT116, SW48, DLD1, HT29, RKO, LoVo, HCT115, and SW480 human colorectal cancer cell lines and HEK293T cells were maintained in McCoy’s 5A or DMEM supplemented with 10% FBS and 100 U penicillin/streptomycin (GE Healthcare Life Sciences) at 37 °C in a 5% CO_2_ incubator. All cell lines were obtained from ATCC. No mycoplasma contamination was detected.

The RNA sequences of USP14 knock-out RKO cell line are presented in the Supplemental Table 3.

### Cell proliferation, Colony formation, and transwell assays

Tumor phenotype analysis was performed as described previously [25, 26]. For the cell proliferation assay, in brief, 2 × 10^3^ cells/well were plated into 96-well plates. The medium was replaced with 1/10 Cell Counting Kit-8 (Dojindo Laboratories) and incubated at 37 °C for 1 h, followed by measurement at an optical density of 450 nm. For the colony formation assay, 400 cells/well were seeded in six-well plates and cultured for 12 days followed by staining with 0.01% crystal violet for 15 min. For the transwell assay, 4 × 10^4^ cells/well containing medium with 1% FBS were seeded in the transwell inserts (Corning) and placed into 24-well plates with medium containing 40% FBS. After 36 h, cells that had migrated outside the chamber were fixed and stained with 0.01% crystal violet.

### Immunohistochemistry

Colorectal cancer tissue microarrays were purchased from Shanghai Outdo Biotech Company (HColA180Su19). Paraffin-embedded mouse colorectal cancer tissue sections (4 μm) were prepared according to the standard protocol. Protein expression was measured with immune peroxidase and scored by the staining intensity and area. Antibodies against USP14, c-Myc, Cyclin d2, and Mdm2 were used to determine their expression levels.

### RNA isolation for real-time quantitative PCR

The cells were harvested in TRIzol reagent (Takara), and total RNA was extracted according to a standard protocol. cDNA was obtained with a reverse transcription kit according to the manufacturer’s instructions (Takara). The sequences of the primers used in the study are presented in Supplementary Table 1.

### Coimmunoprecipitation and western blot analysis

The transfected cells were lysed in lysis buffer (20 mM Tris-HCl, pH 7.4;150 mM NaCl; and 0.5% Triton X-100) with proteinase and phosphatase inhibitor cocktail (Roche). The supernatant of lysates was incubated with 1–3 µg of the indicated antibody at 4 °C for 12 h followed by the addition of protein A/G magnetic beads (Thermo Fisher Scientific, Inc.). Then magnetic beads, antibody, and target protein form complexes at 4 °C for 2-4 h. The complexes were collected by magnetic stand and added 80 µL of SDS loading buffer at 95 °C for 15 min, which making elution of target proteins from beads. The samples were subjected to western blot analysis. Total protein (20–40 μg) was separated by 8–12% SDS-PAGE and transferred to a polyvinylidene fluoride (PVDF) membrane. The membranes were blocked in 5% nonfat milk in Tris-buffered saline with Tween 20 (TBST) for 1 h before incubation with the desired primary antibodies overnight at 4 °C. Then, the membranes were incubated with secondary antibodies at room temperature for 1 h. Finally, the signals were visualized on a ChemiDoc^TM^ XRS + (Bio-Rad). All the uncropped original western blots, uploaded as Supplemental Material.

### Luciferase assay

HEK293T cells (30% confluence) were seeded into 24-well plates and transfected using Lipofectamine 2000 (Thermo Fisher). One hundred nanograms of luciferase reporter plasmid (contains 34 signaling pathways: ATF2/3/4, Antioxidan, ATF6, Cell Cycle, DNA Damage, ER Stress, GATA, Glucocorticoid, Heavy Metal, Hedgehog, HNF4, Hypoxia, Interferon Type, KLF4, Liver X, MAPK/Erk, MAPK/JNK, MEF2, Myc, Nanog, Notch, NF-kB, Xenobiotic, Wnt, VitaminD, TGFβ, STAT3, SP1, Sox2, Retinoid X, Retinoic Acid, PPAR, PKC/Ca + +, Pax6, Oct4), 5 ng of pRL-CMV together with the indicated gene-expressing plasmids (pHAGE-3×Flag-USP14) or empty vector (pHAGE-3×Flag) were transient transfected into the HEK293T cells in each well. After 36 h, the luciferase reporter experiment was carried out using a dual-specific luciferase assay kit from Promega (Fitchburg, WI, USA).

### Obtaining mice colorectal tissue

After the mice were modeled, they were dissected to obtain colorectal tissue. The tissue was divided into two parts, one part was fixed in 4% Paraformaldehyde for 24 h at 4 °C. Then, the expression level of c-Myc, Cyclin d2, and Mdm2 protein in paraffin-embedded CRC tissues of wild-type and USP14-deficient colorectal cancer model mice were examined by immunohistochemistry and H&E staining. Harvested other tissues were immediately snap-frozen and stored at liquid nitrogen container until RNA and protein extraction. Total proteins of colorectal cancer tissues were extracted with lysis buffer, and the total RNA was extracted with TRIzol® Reagent (Takara).

### Statistical analysis

The experiments were repeated at least three times beside mice experiments. Statistical analyses were performed using GraphPad Prism 8.0 software (GraphPad Prism Software Inc.). The data are expressed as the means ± SEM. Student’s *t*-test and analysis of variance (ANOVA) were performed. *P* < 0.05 was regarded to be indicative of statistical significance.

## Results

### USP14 is ectopically overexpressed in colorectal cancer

**Fig. 1:**
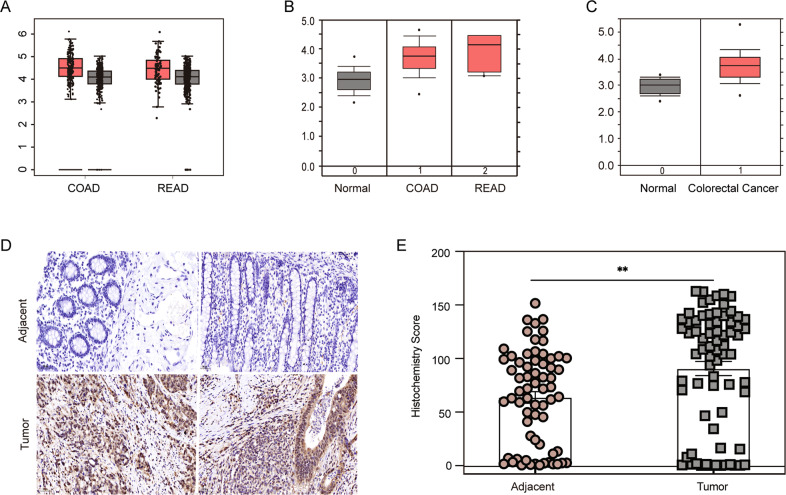
USP14 is upregulated in colorectal cancer. **A** USP14 expression in colon and rectal cancer tissues (red) (*n* = 275 and *n* = 92, respectively) compared with that in relative benign tissues (gray) (*n* = 349 and *n* = 318, respectively) obtained from the GEPIA online tool. **B**, **C** Sabates-Bellver (*n* = 32, *n* = 25 and *n* = 7) and Hong colorectal data (*n* = 12 and *n* = 70) from the Oncomine database indicate USP14 mRNA levels in colorectal cancer patient tissues (red) compared with those in benign tissues (gray). **D** IHC staining for USP14 in colorectal cancer tissue microarrays containing adjacent tissues and normal tissues. **E** Analysis of USP14 expression in colorectal cancer tissue microarrays from 72 tumor and 66 adjacent tissues. COAD, colon adenocarcinoma; READ, rectal adenocarcinoma. The data are presented as the means ± SEM. Statistical significance was analyzed by Student’s *t* test. **p < 0.01.

To determine whether USP14 is upregulated in colorectal cancer, the Gene Expression Profiling Interactive Analysis (GEPIA) web server and Oncomine online tools were used. Figure 1A shows that, according to GEPIA, USP14 RNA levels were significantly upregulated in colon and rectum tumor tissue compared with benign tissue. Sabates-Bellver and Hong colorectal data from the Oncomine database showed a similar pattern of high USP14 expression in colorectal cancer (Fig. 1B, C). To confirm these results, an immunohistochemistry (IHC) experiment was carried out using a tissue microarray containing 72 colorectal cancer patient tissues and 66 adjacent tissues. The IHC results revealed that USP14 was upregulated in colorectal tumor tissues compared with benign tissue (Fig. 1D, E). These data indicated aberrant USP14 expression in colorectal cancer.

### USP14 promotes tumor cell proliferation and migration

**Fig. 2:**
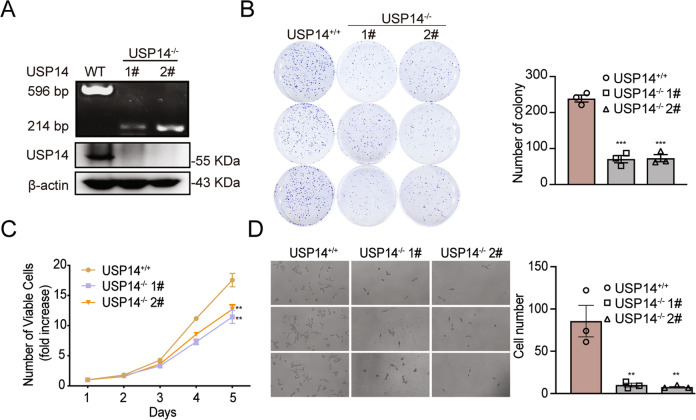
USP14 regulates colorectal cancer cell carcinogenesis. **A** USP14 knockout was identified by genomic PCR and immunoblotting with RKO cells. β-Actin was used as the loading control. **B** The viability of USP14-depleted RKO cells was determined by colony formation experiment. The number of colonies was record (*n* = 3). **C** The cell proliferation ability of USP14-depleted RKO cells was determined by Cell Counting Kit-8 (CCK-8) assay (*n* = 8). **D** The cell migration ability of USP14-depleted RKO cells was determined by transwell experiments (*n* = 3). The data are presented as the means ± SEM. Statistical significance was analyzed by ANOVA. **p < 0.01, ***p < 0.001.

We sought to examine the role of USP14 in colorectal tumor development. The mRNA level of USP14 in various colorectal cancer cell lines, including FHC (normal fetal colonic mucosa cell line), HCT116, SW48, DLD1, HT29, RKO, LoVo, HCT115, and SW480 cells, was tested by real-time PCR (Supplementary Fig. 1A). The SW48 cell line with low USP14 expression was chosen to overexpress flag-tagged USP14 (Supplementary Fig. 1B). Through colony formation assay and cell growth curve generation, we assessed the influence of USP14 on tumor phenotype in SW48 cells in vitro (Supplementary Fig. 1C, D). These tumor phenotype experiments indicated that USP14 overexpression led to considerably increased SW48 number of viable cells (fold increase). To further assess the role of USP14 in colorectal cancer tumorigenesis, we generated USP14-knockdown and USP14-knockout DLD1 and RKO cell lines via CRISPR/Cas9-mediated genomic editing. Genomic PCR, sequencing and Western blot analysis showed the genotype, sequence, and protein level of USP14 in the two cell lines (Fig. 2A and Supplementary Fig. 2A, B). A colony formation assay was performed to determine the viability of these cells, and the results showed decreased colony number and size in the USP14-knockout cell lines (Fig. 2B and Supplementary Fig. 2C). The cell growth curve showed that the targeted USP14-knockout cells significantly inhibited proliferation activity compared with the parental cells (Fig. 2C and Supplementary Fig. 2D). According to the experimental data, we found that the more marked effect on cell proliferation in DLD1 cells where USP14 deletion was less effective than in RKO where it is almost complete. The difference may due to the different background of cell lines’ characteristics, such as the protein level of USP14, proliferation speed and malignancy of cells. Furthermore, transwell assays indicated that USP14 depletion attenuated the migration of RKO cells (Fig. 2D). In summary, USP14 promotes colorectal cancer cell proliferation and migration and enhances colorectal cancer cell viability.

### USP14 regulates the MAPK/JNK signaling cascade

**Fig. 3:**
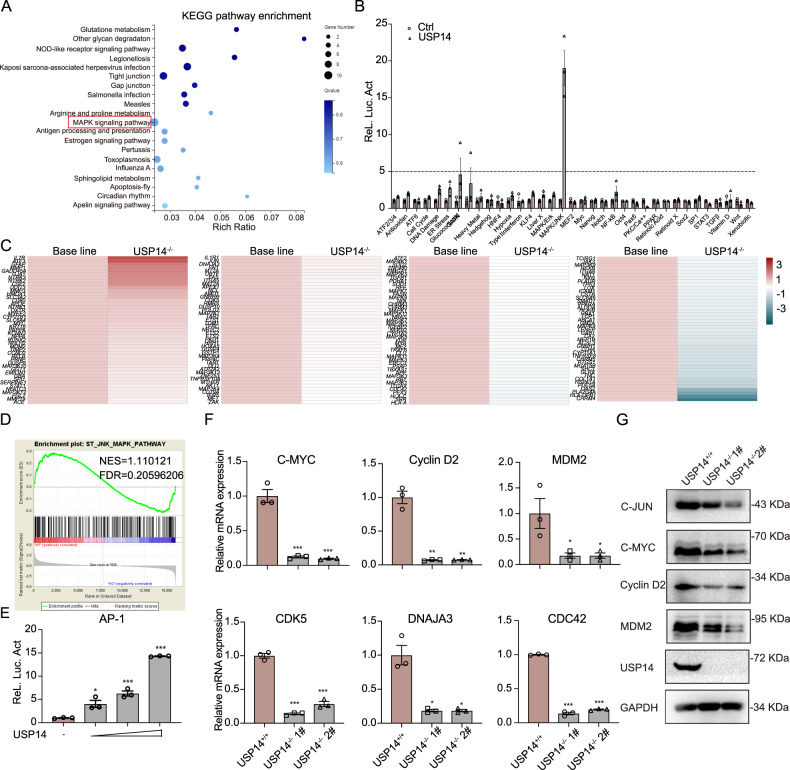
Knockout of USP14 leads to increased MAPK/JNK signaling. **A** KEGG pathway enrichment showed that USP14 regulated the MAPK signaling pathway from the wild-type and USP14-deficient RNA-seq data. **B** USP14 enhanced MAPK/JNK activation as shown by luciferase reporter pathway identification in HEK293T cells (*n* = 3). **C** Heat map analysis of MAPK downstream genes in wild-type and USP14-deficient RKO cells. **D** GSEA of MAPK/JNK target genes in wild-type and USP14-deficient RKO cells. **E** Luciferase assays showing MAPK/JNK activation as indicated by increased amounts of the USP14 plasmid expressed in HEK293T cells (*n* = 3). **F** The mRNA levels of genes with JNK-triggered transcription in USP14-depleted RKO cells were determined by RT-qPCR analysis (*n* = 3). **G** The protein levels of the indicated JNK-targeted genes in USP14-depleted RKO cells were determined by immunoblot analysis. The data are presented as the means ± SEM. Statistical significance was analyzed by ANOVA. *p < 0.05, **p < 0.01, ***p < 0.001.

Next, to dissect the mechanism by which USP14 regulates colorectal tumorigenesis, RNA-seq was performed with USP14-depleted RKO cells (via CRISPR/Cas9-mediated genomic editing). Metabolism- and immune-related genes were largely enriched in Kyoto Encyclopedia of Genomes and Gene (KEGG) pathways. In addition, though the rich ratio is relatively low, the MAPK signaling pathway was among the most enriched pathways (Fig. 3A). On the other hand, USP14 and luciferase reporters were transient overexpressed in 293 T cells to analyze the effect of USP14 on the transcriptional activity of 34 common pathways, which can enhance the activities of DNA Damage, ER stress, GATA, MAPK/JNK, NF-κB pathways. Among them, the activation effect of MAPK/JNK signaling is the most obvious (Fig. 3B). Considering these results in combination, we hypothesized that USP14 regulates the MAPK/JNK signaling pathway. To test this hypothesis, we analyzed differences in MAPK/JNK pathway gene expression in wild-type and USP14-deficient RKO cell lines using heat map and gene set enrichment analysis (GSEA), and the results showed that USP14 deficiency resulted in impaired MAPK/JNK signaling (Fig. 3C, D). Moreover, USP14 expression increased MAPK/JNK luciferase reporter activity in a dose-dependent manner (Fig. 3E). Then, we measured the expression of genes downstream of the classical MAPK/JNK pathway. USP14-deficient RKO cells showed dramatically attenuated mRNA levels of JNK downstream genes, including *C-MYC*, *Cyclin D2*, *MDM2*, *CDK5*, *DNAJA3*, and *CDC42* (Fig. 3F). The measures of the corresponding protein expression levels showed consistent results, with USP14 deficiency resulting in decreased JNK protein levels (Fig. 3G). These results suggested that USP14 regulates the activity of the MAPK/JNK pathway.

### USP14 associates with JNK

**Fig. 4:**
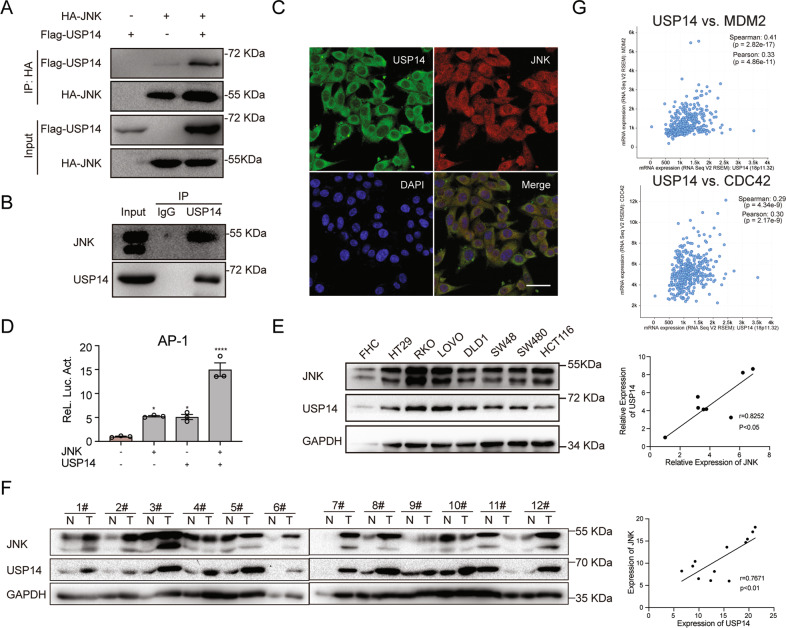
USP14 associates with JNK. **A**, **B** The interaction of exogenous and endogenous USP14 and JNK were examined by immunoprecipitation in HEK293T or RKO cells, respectively. **C** The endogenous colocalization of USP14 (green) and JNK (red) in HEK293T cells was determined by immunofluorescence. DAPI was used to stain nuclei (blue). **D** The synergistic transactivation between USP14 (green) and JNK and AP-1 was assessed by luciferase assays (*n* = 3). **E** USP14 is upregulated and positively correlates with JNK in various colorectal cancer cell lines. **F** USP14 is upregulated and positively correlates with JNK in colorectal cancer specimens. **G** The positive correlation between USP14 and JNK target genes (MDM2 and CDC42) from the cBioPortal online tool. Bar, 50 µm. N normal tissues. T tumor tissues. The data are presented as the means ± SEM. Statistical significance was analyzed by ANOVA. ****p < 0.001.

The MEKK/MKK/JNK signaling cascade is essential for MAPK/JNK activation. Therefore, we evaluated the interaction between USP14 and these proteins. First, we overexpressed the plasmids Flag-USP14 and HA-JNK in 293 T cells and detected the relationship between USP14 and JNK by co-immunoprecipitation experiments. The result showed that JNK interacted with USP14 when JNK and USP14 were co-expressed in 293 T cells (Fig. 4A). To verify the interplay between endogenous USP14 and JNK in colorectal cancer cells, the cell line RKO with higher USP14 level was selected for immunoprecipitation (IP) analysis. The result showed that USP14 strongly binds endogenous JNK also in pathological conditions (Fig. 4B). Furthermore, immunofluorescence analysis was carried out to examine colocalization. The results showed that USP14 mainly colocalized with JNK in the cytosol (Fig. 4C and Supplementary Fig. 3A). AP-1 is a transcription factor of the JNK signaling pathway. After JNK is activated by phosphorylation, it enters the nucleus to activate AP-1, which in turn promotes the binding of AP-1 to the responsive element (TGAGTCAG) and the transcription of downstream genes. Therefore, we overexpressed JNK and USP14 in 293 T cells, respectively, and synergistic activation of AP-1 transcription was tested between USP14 and JNK using a luciferase reporter assay (Fig. 4D). To explore the association of USP14 and JNK considering that USP14 is a DUB, protein levels were examined in both colorectal cell lines and patient tissue specimens. The results showed that USP14 expression was upregulated and positively correlated with JNK expression in various colorectal cell lines and patient tumor tissues (Fig. 4E, F). Correspondingly, the mRNA levels of the JNK downstream genes *MDM2* and *CDC42* also showed a strong correlation with USP14 (Fig. 4G). In summary, these data suggest that JNK interacts with and is closely related to USP14.

### USP14 deubiquitinates and increases JNK stability

**Fig. 5:**
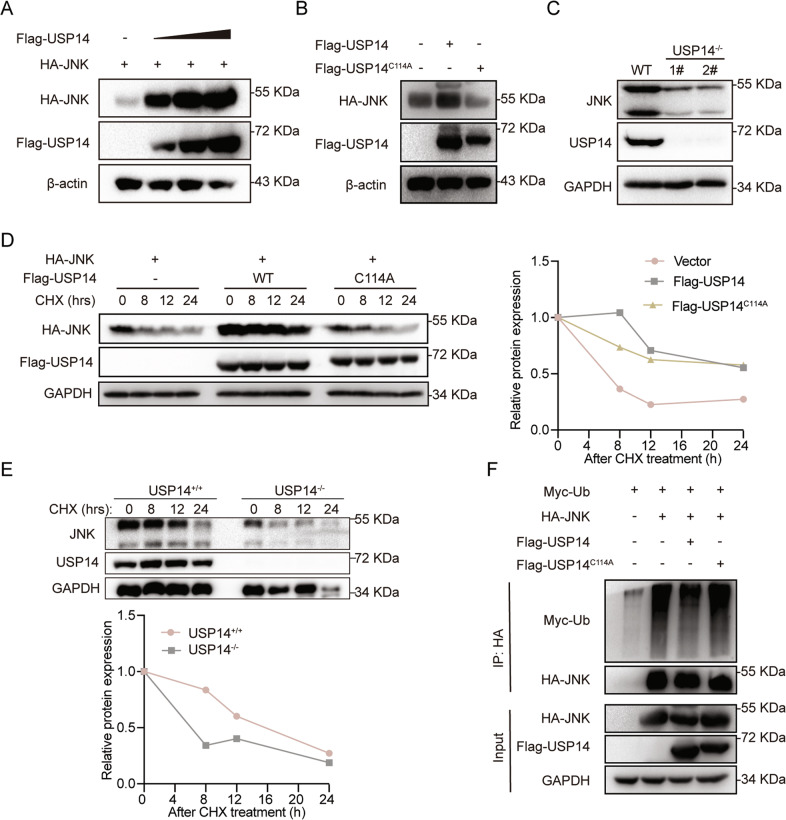
USP14 stabilizes and deubiquitinates JNK. **A** The protein level is positively correlated with HA-JNK and increasing Flag-USP14 protein (0, 400 ng, 600 ng, and 800 ng) in HEK293T cells by immunoblot assay. **B** The effect of USP14 or the USP14^C114A^ mutant on JNK protein levels in HEK293T cells. **C** The protein level of JNK in USP14-deficient RKO cells. **D** The stability of JNK in the presence of USP14 or the USP14^C114A^ mutant after treatment with CHX (50 μg/ml) at the indicated time points. **E** The stability of JNK in the absence of USP14 after treatment with CHX (50 μg/ml) at the indicated time points. **F** JNK ubiquitination level in the presence of USP14 or the USP14^C114A^ mutant.

Because USP14 is a DUB, its deubiquitinating activity is crucial to its function. The active site of USP14, Cys114, was replaced with an Ala residue to acquire a Ub hydrolase-deficient mutant (USP14^C114A^) [27]. Then, we assessed the extent to which JNK stability is regulated by USP14 by co-expressing HA-JNK and Flag-USP14 (0, 400 ng, 600 ng, and 800 ng) in the 293 T cells. A Western blot analysis revealed that USP14 forced the expression of JNK in a dose-dependent manner (Fig. 5A). To determine the effect of USP14 DUB activity on JNK stability, we overexpressed USP14 or the USP14^C114A^ mutant, and the results showed that the DUB activity of USP14 is required for JNK stability (Fig. 5B). Next, we assessed the effect of USP14 deficiency on the protein levels of JNK. The results showed that USP14 ablation decreased the JNK levels in the USP14-deficient cell line (Fig. 5C). A cycloheximide chase (CHX) assay was performed to verify that JNK protein levels are regulated by USP14 over time. The protein half-life of JNK was obviously prolonged in the presence of USP14 compared with the USP14^C114A^ mutant (Fig. 5D). Accordingly, USP14 deficiency decreased the JNK protein half-life (Fig. 5E). Finally, we sought to determine whether the ubiquitination level of JNK was regulated by USP14. The results showed that the ubiquitin level of JNK was sharply reduced by USP14 but not by the USP14^C114A^ mutant, indicating that the JNK ubiquitination level was dependent on USP14 DUB activity (Fig. 5F). Collectively, these data suggest that USP14 deubiquitinates and stabilizes the JNK protein.

### USP14-knockout inhibits tumor formation in vivo

**Fig. 6:**
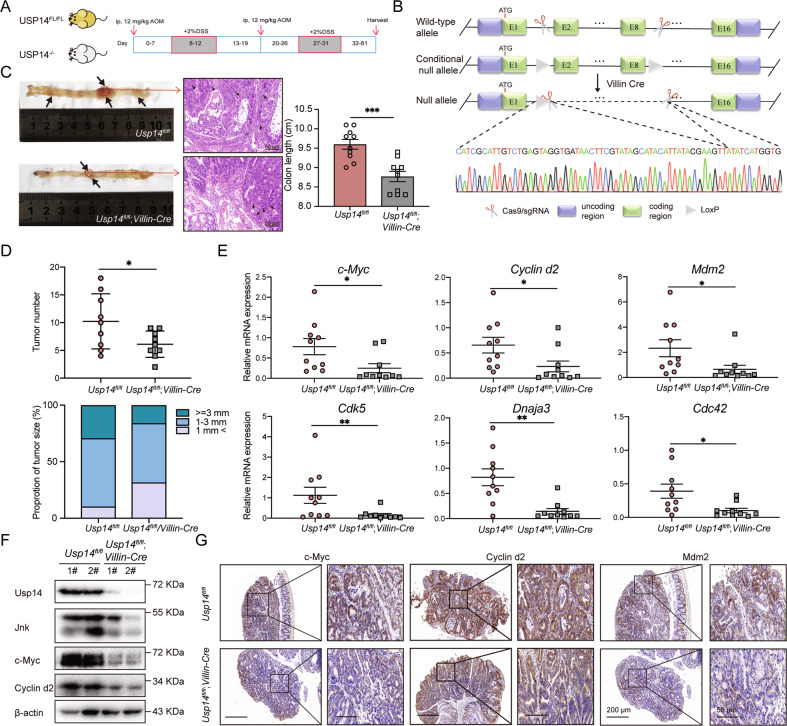
Genetic depletion of USP14 alleviates tumorigenesis in vivo. **A** A schematic of the AOM/DSS-induced colorectal cancer model. **B** The USP14 genomic sequence after CRISPR/Cas9 edit. **C** H&E staining and colon length between the wild-type and USP14-deficient colorectal cancer model mice. The black arrows show location of the tumors. **D** The number and size of tumors in the wild-type and USP14-deficient colorectal cancer model mice. **E** The mRNA levels of the indicated JNK target genes in the wild-type and USP14-deficient colorectal cancer model mice (*n* = 10). **F** The protein levels of the indicated JNK target genes in the wild-type and USP14-deficient colorectal cancer model mice. **G** Image of IHC staining for c-Myc, Cyclin d2, and Mdm2 in the tissues of wild-type and USP14-deficient colorectal cancer model mice. The data are presented as the means ± SEM. Statistical significance was analyzed by Student’s *t* test. *p < 0.05, **p < 0.01, ***p < 0.001, ***p < 0.0001.

To further clarify the tumorigenic role of *Usp14* in colorectal cancer in vivo, we generated intestinal-specific *Usp14-*deleted mice (*Usp14*^*fl/fl*^*;Villin-Cre*) using *Usp14*^*fl/fl*^ conditional knockout mice crossed with intestinal-specific Villin-Cre mice. An AOM/DSS-induced colorectal cancer model was established to assess the effect of intestinal-specific *Usp14* depletion on tumorigenesis (Fig. 6A) [28]. The knockout strategy and the confirmation of *Usp14* deficiency by genomic PCR sequencing and Western blotting are shown in Fig. 6B and Supplementary Fig. 4A. The body weights of the wild-type mice were lower than those of the *Usp14*^*fl/fl*^*;Villin-Cre* mice in the AOM/DSS-induced colorectal cancer model (Supplementary Fig. 4B). At the end of the experiment, hematoxylin and eosin (H&E) staining was performed to verify tumor development, and *Usp14*^*fl/fl*^*;Villin-Cre* mice displayed a notable decrease in tumor number and size compared with wild-type mice (Fig. 6C, D). These results suggested that *Usp14* knockout alleviated AOM/DSS-induced colorectal tumorigenesis in vivo. Moreover, we analyzed the mRNA levels of downstream genes in the JNK pathway, and the quantitative PCR (qPCR) results showed dramatically reduced mRNA levels of genes in the colorectal-specific *Usp14-*deficient mice (Fig. 6E). The protein levels of Jnk, c-Myc, and Cyclin d2 was also decreased in *Usp14* colorectal-specific deficient mice, which is consistent with the IHC staining results (Fig. 6F, G). In conclusion, *Usp14* tends to facilitate colorectal cancer progression by stabilizing JNK and subsequently promoting pathway activation.

**Fig. 7:**
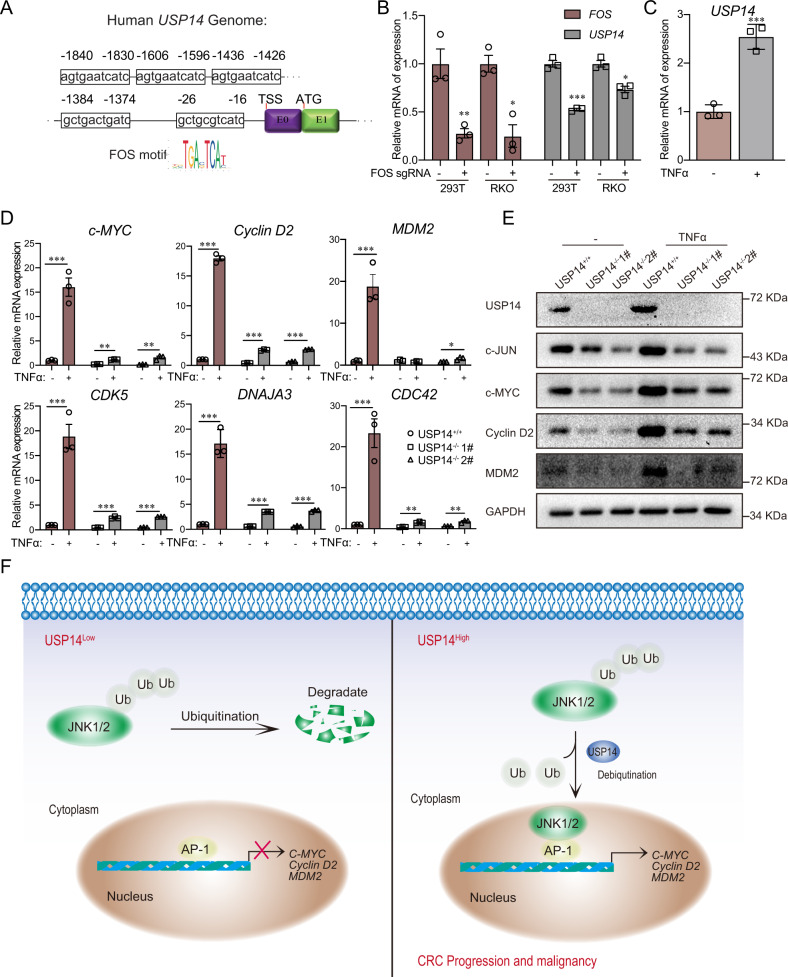
USP14 is regulated by TNF-α and required for TNF-α-induction of JNK downstream genes. **A** A schematic of the FOS motif in the human *USP14* promoter. **B** The mRNA level of USP14 in FOS-knockdown cells and wild-type cells (*n* = 3). **C** The mRNA expression of *USP14* induced by TNF-α. **D** The mRNA expression of TNF-α-induced JNK target genes in wild-type and USP14-deficient RKO cells (*n* = 3). **E** The protein levels of USP14, c-JUN, c-MYC, Cyclin D2 and MDM2 in wild-type and USP14-deficient RKO cells. **F** Working model showing how USP14 functions as an oncogene to promote tumorigenesis through JNK. The data are presented as the means ± SEM. Statistical significance was analyzed by ANOVA or Student’s *t* test. *p < 0.05, **p < 0.01, ***p < 0.001.

To evaluate whether the JNK-USP14 axis is part of a feedback loop, we used the online tool JASPAR to identify FOS, a JNK-regulated downstream transcription factor, which binds sites in the *USP14* promoter. The results showed that multiple FOS-binding sites were located in the *USP14* promoter, which indicated that FOS may control the transcriptional regulation of *USP14* (Fig. 7A). To confirm this, we have knocked down FOS in 293 T and RKO cells, and the qPCR results showed mRNA level of USP14 was downregulated in *FOS-*knockdown cells compared with wild-type cells (Fig. 7B). Because JNK pathway activation is closely related to the inflammatory response, we activated the JNK pathway using the TNF-α cytokine. The TNF-α-triggered JNK pathway upregulated the mRNA level of USP14 (Fig. 7C). In addition, *USP14* deficiency dramatically repressed TNF-α-induced downstream gene activation (Fig. 7D). Similarly, TNF-α-induced upregulation of downstream proteins was impaired in *USP14-*knockout cells compared with wild-type cells (Fig. 7E), which indicated that USP14 is required for TNF-α-induced JNK signaling. In summary, these data support the idea that USP14 promotes colorectal tumorigenesis through a JNK-/USP14-positive feedback loop (Fig. 7F).

## Discussion

DUB family members maintain ubiquitin homeostasis by removing ubiquitin from target proteins, thus participating in the development of multiple diseases, such as cancer. As a DUB family member, USP14 is aberrantly expressed in various kinds of cancer. However, the role of USP14 in colorectal cancer remains to be determined. First, we analyzed USP14 expression in The Cancer Genome Atlas (TCGA) and Oncomine datasets, which showed that USP14 was upregulated in colorectal cancer patients. Our results from the IHC staining experiments with a tissue microarray supported this result. To determine USP14 function in colorectal carcinogenesis, we examined the phenotype using USP14-overexpressing or USP14-deficient colorectal cancer cell lines. The results suggest that USP14 is a pivotal driver of colorectal cancer development and progression. Shinji et al. reported that USP14 was detected at high a level in the cytoplasm of colorectal cancer patients and that this finding correlated with pathological stage as well as lymph node and liver metastases. These results are consistent with the authors’ conclusion, but we did not find a correlation between USP14 expression and the pathological stage or overall survival rate of patients [29]. The difference in studies may be due to differences in the patient cohorts. Similarly, we generated colorectal-specific USP14-deficient mice. The AOM/DSS-induced colorectal cancer model verified the oncogenic role of USP14, which extended the evidence showing that USP14 is an oncogene in vivo.

We next set out to dissect the mechanism. RNA sequencing and luciferase pathway screening pointed to the MAPK/JNK pathway. We further verified the effects of USP14 on this pathway at the mRNA and protein levels through MAPK/JNK target genes. Vaden *et al*. reported that USP14 regulated neuromuscular junctions through its effects on JNK signaling [13], which is consistent with our results obtained through a different system. However, the specific mechanism and target were not clarified by these analyses. In co-IP experiments, we found that USP14 interacted with JNK and deubiquitinated and stabilized the JNK protein. Finally, the AOM/DSS-induced colorectal cancer model was used in experiments that further demonstrated the regulation of MAPK/JNK signaling by USP14. FASN, RNF168, and Ku70 were found to be substrates of USP14 [17, 30, 31]. In this study, we revealed a new substrate of USP14 and clarified its function. These findings contribute to the understanding of the carcinogenic role of USP14. MAPK signaling is extensively involved in cancer development and progression. Thus, we tested the hypothesis that USP14 promotes colorectal cancer carcinogenesis by targeting JNK for stabilization.

TNF-α triggered the activity of JNK [32, 33]. Although more solid evidence needs to be obtained, our preliminary results indicate that USP14 expression is induced by TNF-α and is required for TNF-α-triggered JNK activation. USP14 has been reported to regulate the production of TNF-α; [34, 35] therefore, our study supports the formation of a TNF-α/JNK/USP14 feedback loop. In addition, USP14 has been considered a therapeutic target for the treatment of neurodegenerative diseases, and selective inhibitors of USP14 were developed in 2010 [36–39]. Further studies need to be performed to evaluate whether this treatment strategy works against tumor development and progression through specific inhibitors selectively targeting USP14.

In summary, we found that USP14 is upregulated and positively associated with JNK in colorectal cancer. As an oncogene, USP14 deubiquitinates and mediates the stability of JNK, which in turn amplifies MAPK/JNK signaling and potentiates colorectal carcinogenesis in vivo and in vitro. Our study reveals a new carcinogenic mechanism of USP14, which may be a potential therapeutic target for colorectal cancer.

## Supplementary information

Supplementary Figure 1

Supplementary Figure 2

Supplementary Figure 3

Supplementary Figure 4

Supplementary Figure Legends

Supplementary Table 1

Supplementary Table 2

Supplementary Table 3

uncropped WB-1

uncropped WB-2

uncropped WB-3

uncropped WB-4

uncropped WB-5

aj-checklist

## Data Availability

The experimental data sets generated and/or analyzed during the current study are available from the corresponding author upon reasonable request.

## References

[1] Kim EK, Choi EJ (2015). Compromised MAPK signaling in human diseases: an update. Arch Toxicol.

[2] Lawrence MC, Jivan A, Shao C, Duan L, Goad D, Zaganjor E (2008). The roles of MAPKs in disease. Cell Res.

[3] Kassouf T, Sumara G (2020). Impact of conventional and atypical MAPKs on the development of metabolic diseases. Biomolecules.

[4] Wang X, Destrument A, Tournier C (2007). Physiological roles of MKK4 and MKK7: insights from animal models. Biochim Biophys Acta.

[5] Tournier C, Dong C, Turner TK, Jones SN, Flavell RA, Davis RJ (2001). MKK7 is an essential component of the JNK signal transduction pathway activated by proinflammatory cytokines. Genes Dev.

[6] Kumar A, Singh UK, Kini SG, Garg V, Agrawal S, Tomar PK (2015). JNK pathway signaling: a novel and smarter therapeutic targets for various biological diseases. Future Med Chem.

[7] Sancho R, Nateri AS, de Vinuesa AG, Aguilera C, Nye E, Spencer-Dene B (2009). JNK signalling modulates intestinal homeostasis and tumourigenesis in mice. EMBO J.

[8] Zeke A, Misheva M, Remenyi A, Bogoyevitch MA (2016). JNK signaling: regulation and functions based on complex protein-protein partnerships. Microbiol Mol Biol Rev.

[9] Ha J, Kang E, Seo J, Cho S (2019). Phosphorylation dynamics of JNK signaling: effects of dual-specificity phosphatases (DUSPs) on the JNK pathway. Int J Mol Sci.

[10] Davis RJ (2000). Signal transduction by the JNK group of MAP kinases. Cell.

[11] Li P, Huang P, Li X, Yin D, Ma Z, Wang H (2018). Tankyrase mediates K63-linked ubiquitination of JNK to confer stress tolerance and influence lifespan in Drosophila. Cell Rep.

[12] van Wijk SJ, Fulda S, Dikic I, Heilemann M (2019). Visualizing ubiquitination in mammalian cells. EMBO Rep.

[13] Vaden JH, Bhattacharyya BJ, Chen PC, Watson JA, Marshall AG, Phillips SE (2015). Ubiquitin-specific protease 14 regulates c-Jun N-terminal kinase signaling at the neuromuscular junction. Mol Neurodegener.

[14] Min Y, Lee S, Kim MJ, Chun E, Lee KY (2017). Ubiquitin-specific protease 14 negatively regulates toll-like receptor 4-mediated signaling and autophagy induction by inhibiting ubiquitination of TAK1-binding protein 2 and beclin 1. Front Immunol.

[15] Liu N, Kong T, Chen X, Hu H, Gu H, Liu S (2017). Ubiquitin-specific protease 14 regulates LPS-induced inflammation by increasing ERK1/2 phosphorylation and NF-kappaB activation. Mol Cell Biochem.

[16] Li H, Zhao Z, Ling J, Pan L, Zhao X, Zhu H (2019). USP14 promotes K63-linked RIG-I deubiquitination and suppresses antiviral immune responses. Eur J Immunol.

[17] Liu B, Jiang S, Li M, Xiong X, Zhu M, Li D (2018). Proteome-wide analysis of USP14 substrates revealed its role in hepatosteatosis via stabilization of FASN. Nat Commun.

[18] Wang Y, Wang J, Zhong J, Deng Y, Xi Q, He S (2015). Ubiquitin-specific protease 14 (USP14) regulates cellular proliferation and apoptosis in epithelial ovarian cancer. Med Oncol.

[19] Chen X, Wu J, Chen Y, Ye D, Lei H, Xu H (2016). Ubiquitin-specific protease 14 regulates cell proliferation and apoptosis in oral squamous cell carcinoma. Int J Biochem Cell Biol.

[20] Liao Y, Liu N, Hua X, Cai J, Xia X, Wang X (2017). Proteasome-associated deubiquitinase ubiquitin-specific protease 14 regulates prostate cancer proliferation by deubiquitinating and stabilizing androgen receptor. Cell Death Dis.

[21] Han KH, Kwak M, Lee TH, Park MS, Jeong IH, Kim MJ (2019). USP14 inhibition regulates tumorigenesis by inducing autophagy in lung cancer in vitro. Int J Mol Sci.

[22] Jung H, Kim BG, Han WH, Lee JH, Cho JY, Park WS (2013). Deubiquitination of dishevelled by Usp14 is required for Wnt signaling. Oncogenesis.

[23] Huang G, Li L, Zhou W (2015). USP14 activation promotes tumor progression in hepatocellular carcinoma. Oncol Rep.

[24] Xia X, Huang C, Liao Y, Liu Y, He J, Guo Z (2019). Inhibition of USP14 enhances the sensitivity of breast cancer to enzalutamide. J Exp Clin Cancer Res.

[25] Zhang MH, Zhang HH, Du XH, Gao J, Li C, Shi HR (2020). UCHL3 promotes ovarian cancer progression by stabilizing TRAF2 to activate the NF-kappaB pathway. Oncogene.

[26] Zhang HH, Li C, Ren JW, Liu L, Du XH, Gao J (2021). OTUB1 facilitates bladder cancer progression by stabilizing ATF6 in response to endoplasmic reticulum stress. Cancer Sci.

[27] Chadchankar J, Korboukh V, Conway LC, Wobst HJ, Walker CA, Doig P (2019). Inactive USP14 and inactive UCHL5 cause accumulation of distinct ubiquitinated proteins in mammalian cells. PLoS ONE.

[28] De Robertis M, Massi E, Poeta ML, Carotti S, Morini S, Cecchetelli L (2011). The AOM/DSS murine model for the study of colon carcinogenesis: From pathways to diagnosis and therapy studies. J Carcinog.

[29] Shinji S, Naito Z, Ishiwata S, Ishiwata T, Tanaka N, Furukawa K (2006). Ubiquitin-specific protease 14 expression in colorectal cancer is associated with liver and lymph node metastases. Oncol Rep.

[30] Sharma A, Alswillah T, Singh K, Chatterjee P, Willard B, Venere M (2018). USP14 regulates DNA damage repair by targeting RNF168-dependent ubiquitination. Autophagy.

[31] Sharma A, Alswillah T, Kapoor I, Debjani P, Willard B, Summers MK (2020). USP14 is a deubiquitinase for Ku70 and critical determinant of non-homologous end joining repair in autophagy and PTEN-deficient cells. Nucleic Acids Res.

[32] Kant S, Swat W, Zhang S, Zhang ZY, Neel BG, Flavell RA (2011). TNF-stimulated MAP kinase activation mediated by a Rho family GTPase signaling pathway. Genes Dev.

[33] Mong PY, Petrulio C, Kaufman HL, Wang Q (2008). Activation of Rho kinase by TNF-alpha is required for JNK activation in human pulmonary microvascular endothelial cells. J Immunol.

[34] Li H, Quan J, Zhao X, Ling J, Chen W (2021). USP14 negatively regulates RIG-I-mediated IL-6 and TNF-alpha production by inhibiting NF-kappaB activation. Mol Immunol.

[35] Mialki RK, Zhao J, Wei J, Mallampalli DF, Zhao Y (2013). Overexpression of USP14 protease reduces I-kappaB protein levels and increases cytokine release in lung epithelial cells. J Biol Chem.

[36] Adelakun N, Obaseki I, Adeniyi A, Fapohunda O, Obaseki E, Omotuyi O (2022). Discovery of new promising USP14 inhibitors: computational evaluation of the thumb-palm pocket. J Biomol Struct Dyn.

[37] Wang Y, Jiang Y, Ding S, Li J, Song N, Ren Y (2018). Small molecule inhibitors reveal allosteric regulation of USP14 via steric blockade. Cell Res.

[38] Lundgren S, Odrzywol E (2018). USP14 inhibitors as potential anticancer agents. Future Med Chem.

[39] Lee BH, Finley D, King RW (2012). A high-throughput screening method for identification of inhibitors of the deubiquitinating enzyme USP14. Curr Protoc Chem Biol.

