# Supplementary figures and images for: USP14 promotes colorectal cancer progression by targeting JNK for stabilization
Source: Cell Death Dis. 2023 Jan 24;14(1):56. doi: 10.1038/s41419-023-05579-5 (PMC9873792; doi:10.1038/s41419-023-05579-5)

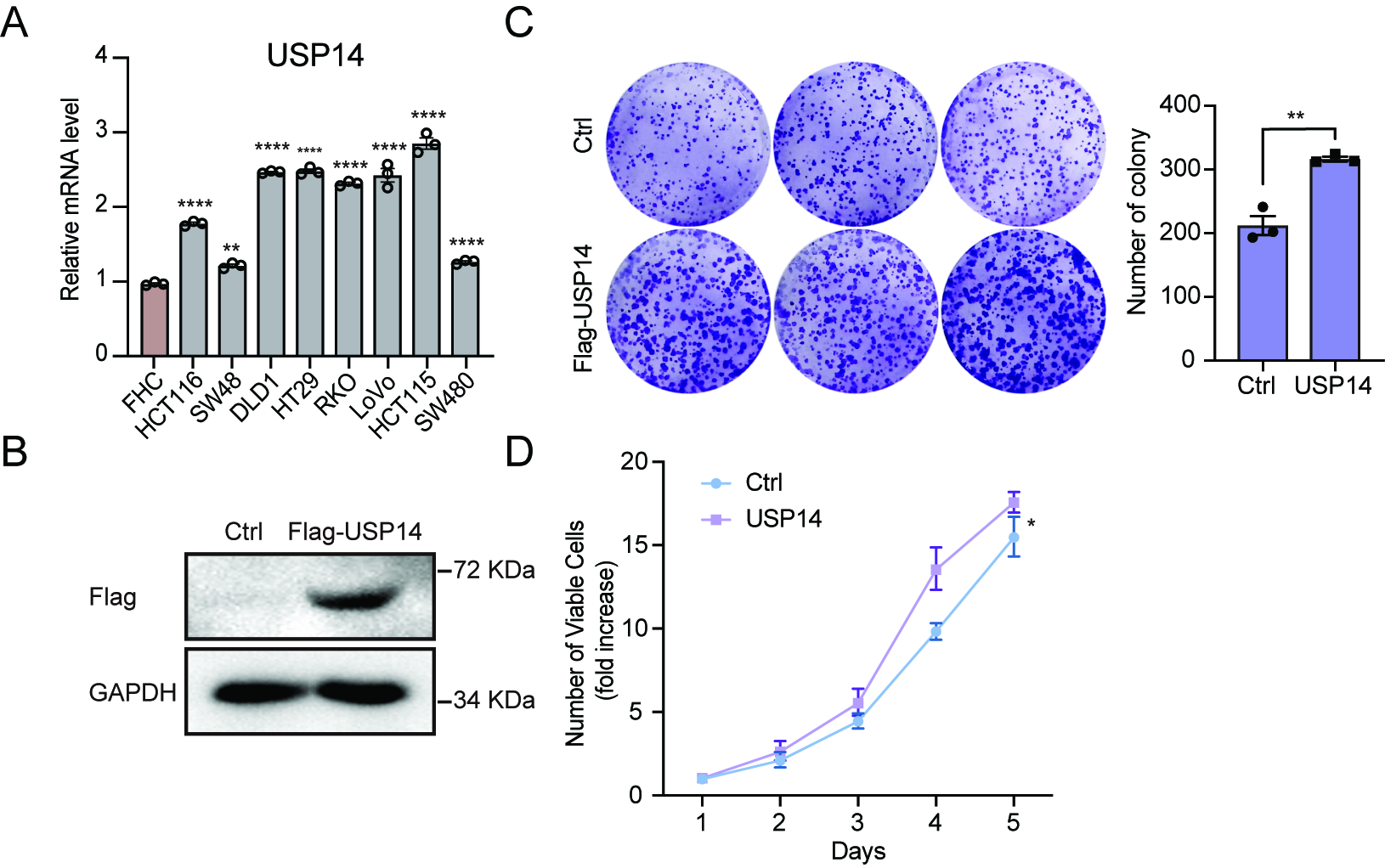

Supplement: Supplementary file 1 — Supplementary Figure 1 [file 41419_2023_5579_MOESM1_ESM.tif]

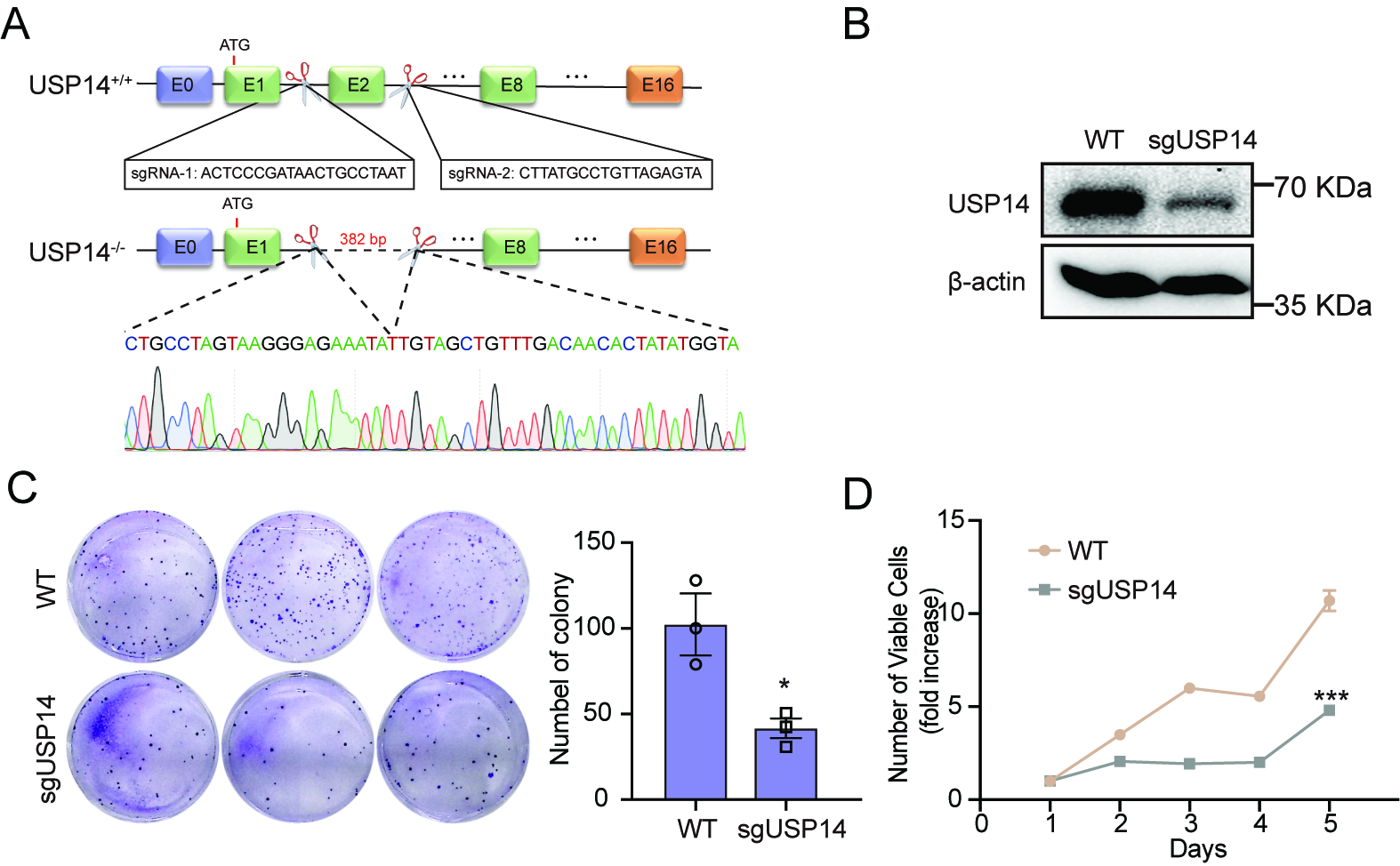

Supplement: Supplementary file 2 — Supplementary Figure 2 [file 41419_2023_5579_MOESM2_ESM.tif]

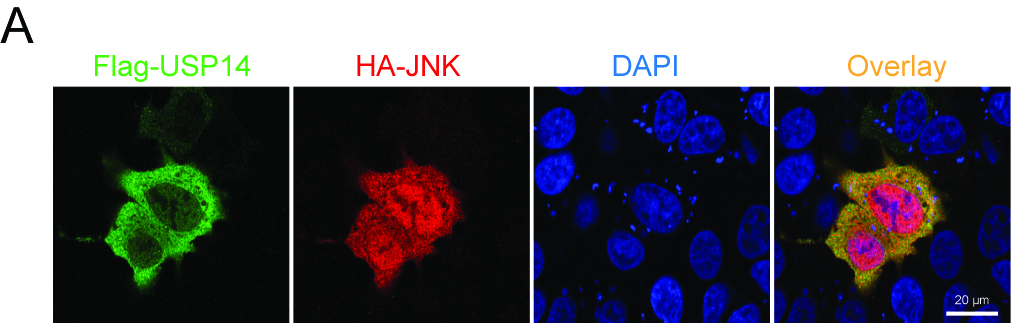

Supplement: Supplementary file 3 — Supplementary Figure 3 [file 41419_2023_5579_MOESM3_ESM.tif]

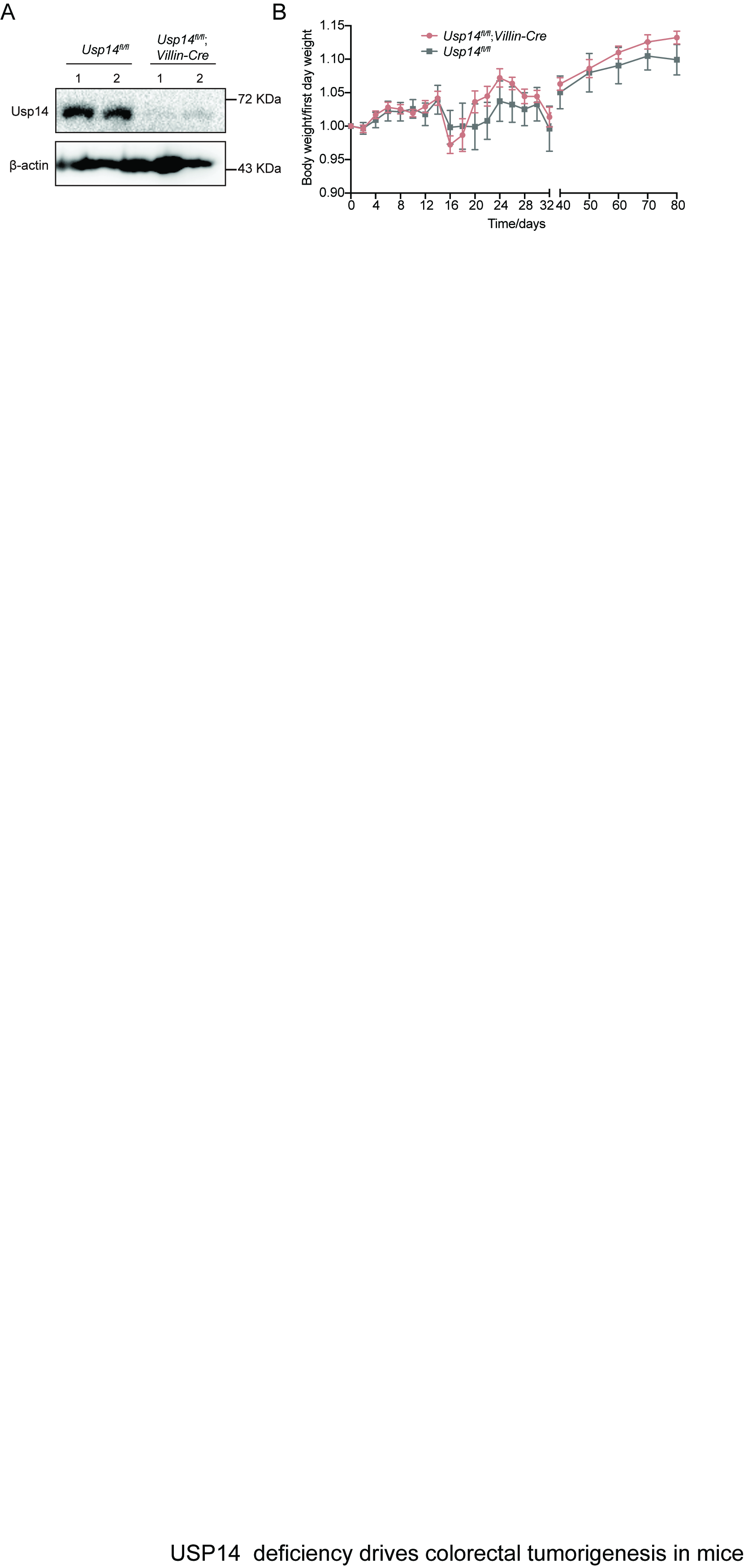

Supplement: Supplementary file 4 — Supplementary Figure 4 [file 41419_2023_5579_MOESM4_ESM.tif]

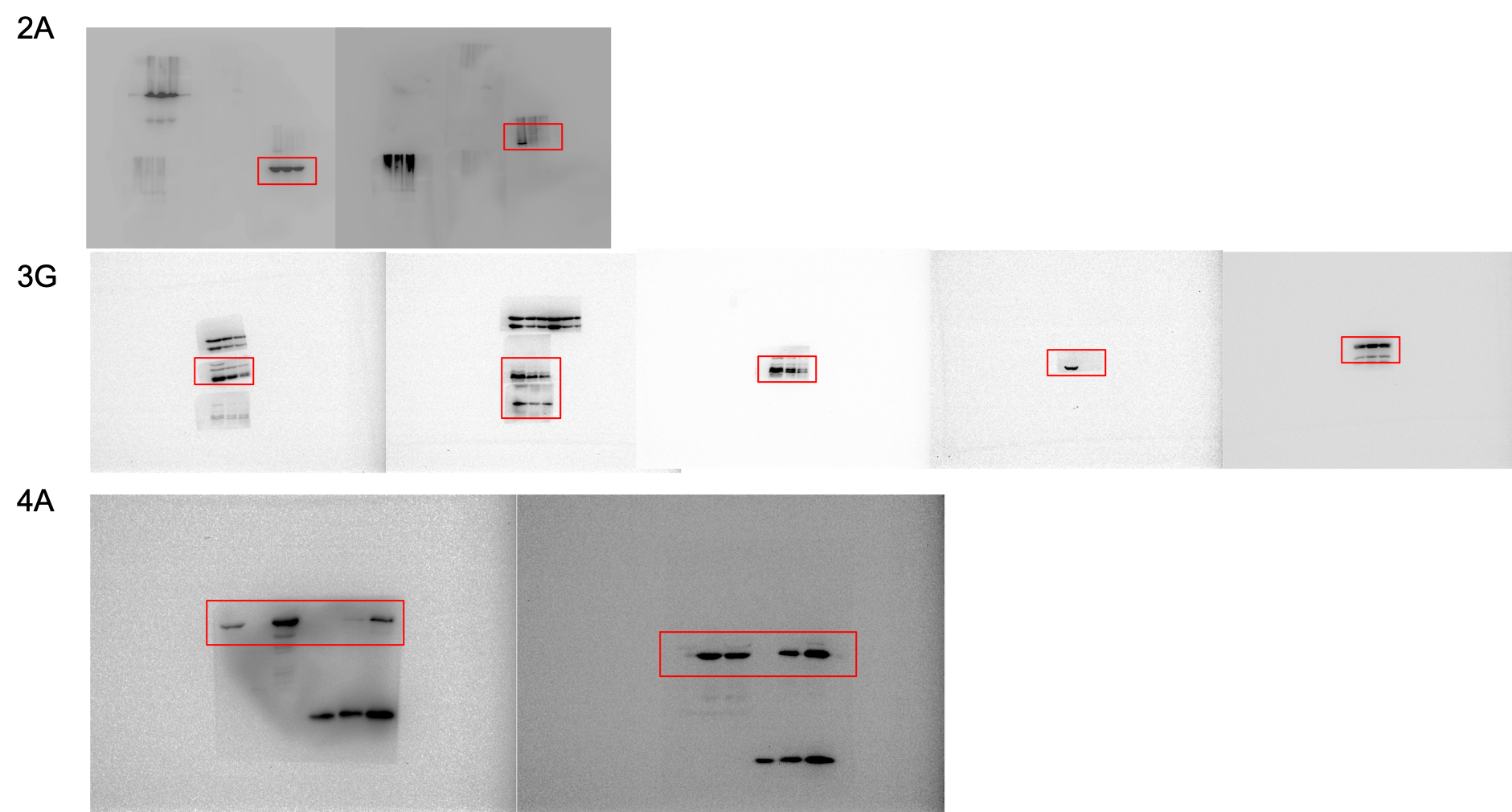

Supplement: Supplementary file 9 — uncropped WB-1 [file 41419_2023_5579_MOESM9_ESM.png]

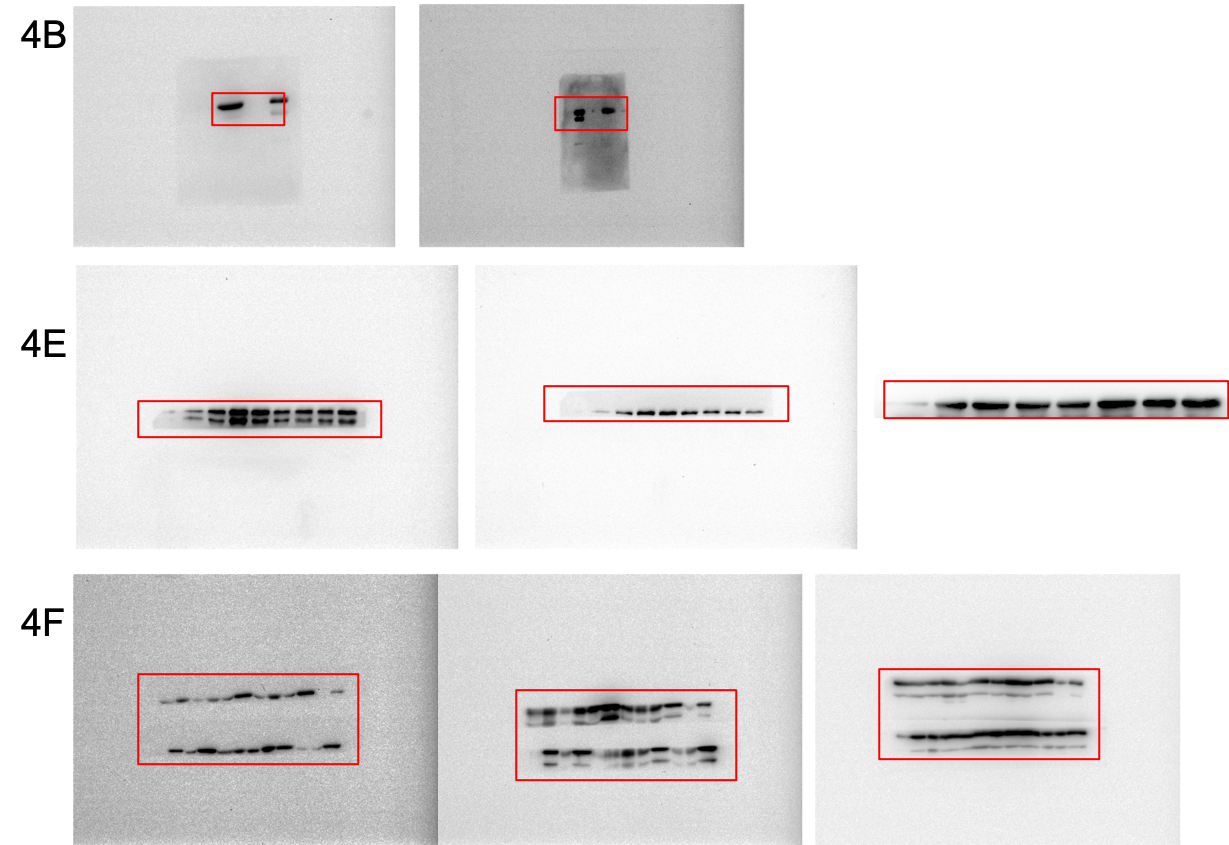

Supplement: Supplementary file 10 — uncropped WB-2 [file 41419_2023_5579_MOESM10_ESM.png]

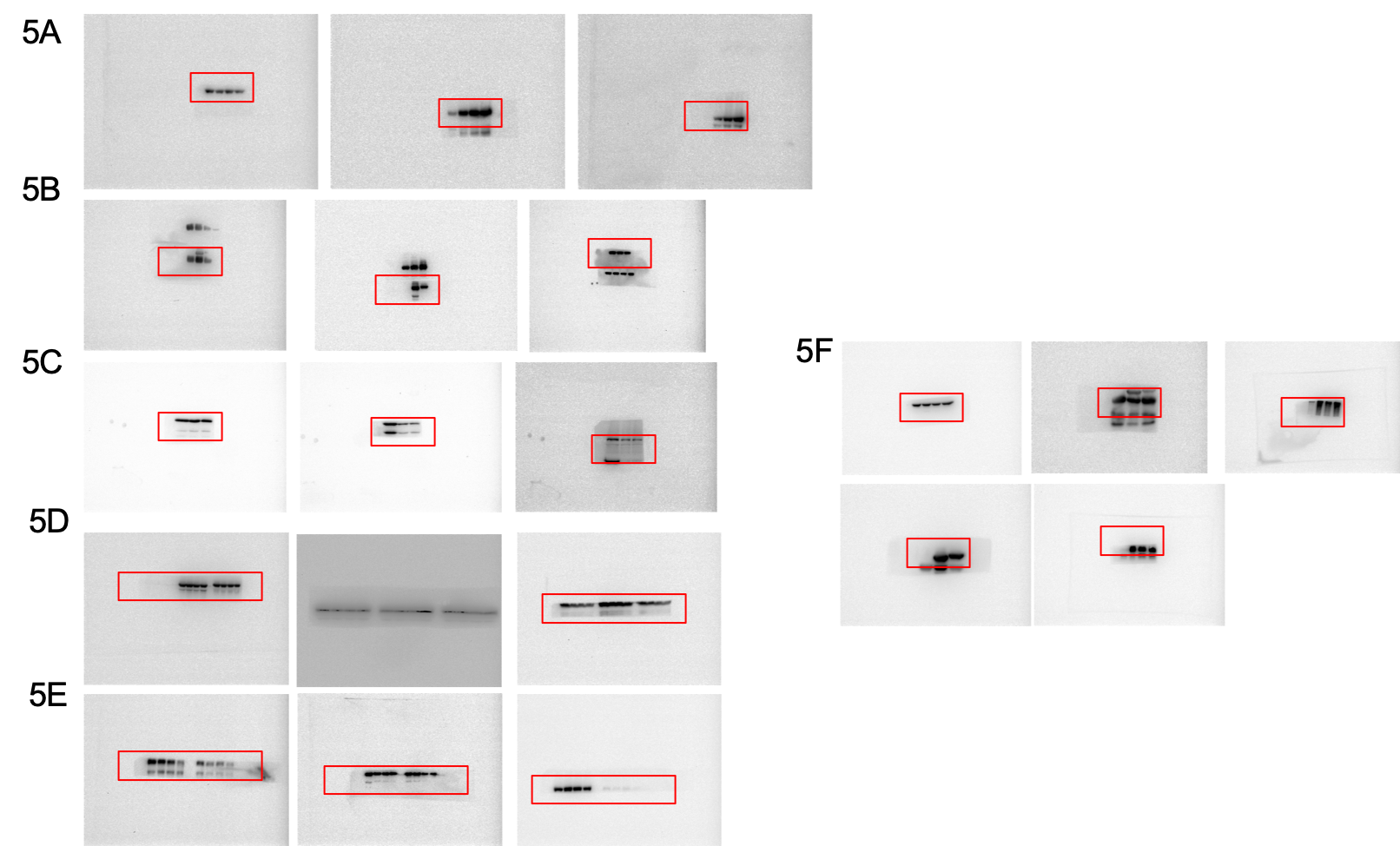

Supplement: Supplementary file 11 — uncropped WB-3 [file 41419_2023_5579_MOESM11_ESM.png]

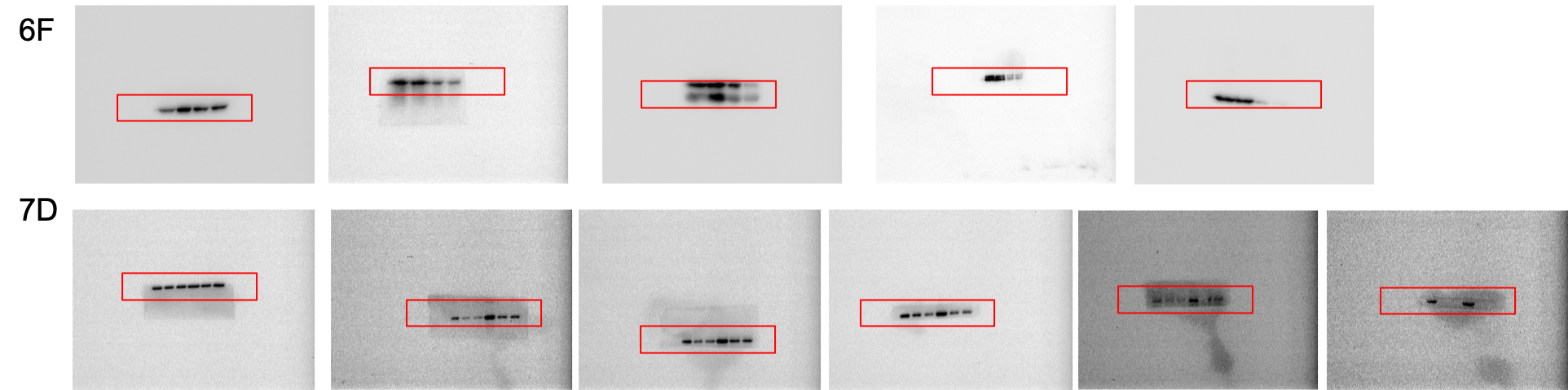

Supplement: Supplementary file 12 — uncropped WB-4 [file 41419_2023_5579_MOESM12_ESM.png]

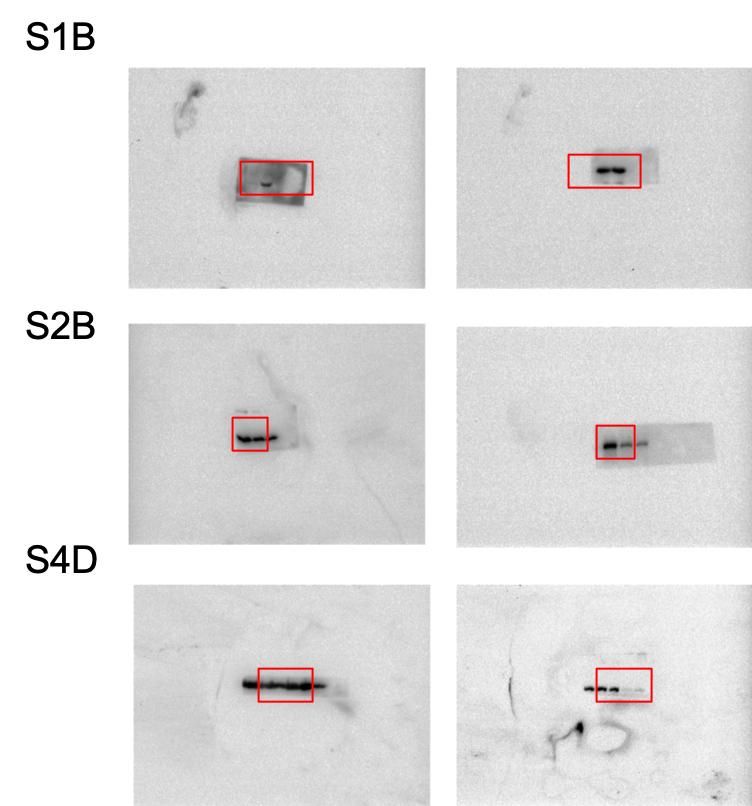

Supplement: Supplementary file 13 — uncropped WB-5 [file 41419_2023_5579_MOESM13_ESM.png]
