# Supplementary material for: USP14 promotes colorectal cancer progression by targeting JNK for stabilization
Source: Cell Death Dis. 2023 Jan 24;14(1):56. doi: 10.1038/s41419-023-05579-5 (PMC9873792; doi:10.1038/s41419-023-05579-5)
Supplement: Supplementary file 5 — Supplementary Figure Legends [file 41419_2023_5579_MOESM5_ESM.docx]

**Supplementary Figure 1. USP14 regulates colorectal carcinogenesis**

(A) RT-qPCR assay was performed with USP14 mRNA level in FHC, HCT116, SW48, DLD1, HT29, RKO, LoVo, HCT115, and SW480 colorectal cancer cell lines. (B) Immunoblot analysis confirmed stable USP14 protein expression in SW48 cells. GAPDH was used as the loading control. (C) The viability of USP14-overexpressing SW48 cells was determined by colony formation experiments. The number of colonies was recorded (n=3). (D) The proliferation of USP14-overexpressing SW48 cells was tested by CCK-8 assay (n=8). The data are presented as the mean ± SEM. Statistical significance was analyzed by ANOVA or Student’s *t*-test. **p* < 0.05, ***p* < 0.01, *****p* < 0.0001.

**Supplementary Figure 2. USP14 regulates colorectal carcinogenesis**

(A) The genomic sequence of USP14 depletion was determined by sequencing. (B) USP14 knockout in DLD1 cells was identified by immunoblot. β-Actin was used as a loading control. (C) The viability of USP14-depleted DLD1 cells was determined by colony formation experiments. The number of colonies was recorded (n=3). (D) The proliferation ability of USP14-depleted DLD1 cells was tested by CCK-8 assay (n=8). The data are presented as the means ± SEM. Statistical significance was analyzed by ANOVA or Student’s *t*-test. **p* < 0.05, ****p* < 0.001.

**Supplementary Figure 3. USP14 associates with JNK**

(A) The exogenous colocalization between USP14 (green) and JNK (red) was examined by immunofluorescence in HEK293T cells. DAPI stained nuclei stain (blue). Bar, 20 µm.

**Supplementary Figure 4. Genetic depletion of USP14 attenuates tumorigenesis *in vivo*.**

(A) The protein level of USP14 in wild-type and USP14-deficient colorectal cancer model mice. (B) The average body weight of the AOM/DSS-induced colorectal cancer model mice.
