# Supplementary material for: USP14 promotes colorectal cancer progression by targeting JNK for stabilization
Source: Cell Death Dis. 2023 Jan 24;14(1):56. doi: 10.1038/s41419-023-05579-5 (PMC9873792; doi:10.1038/s41419-023-05579-5)
Supplement: Supplementary file 7 — Supplementary Table 2 [file 41419_2023_5579_MOESM7_ESM.docx]

**Supplementary table 2: Downstream genes of MAPK/JNK signaling pathway**

| Gene |
| --- |
| IL7R |
| ATF3 |
| GFAP |
| MMP1 |
| GADD45A |
| NHLH2 |
| RUNX2 |
| NPPA |
| KCNN3 |
| PAPPA |
| KRT18 |
| MYC |
| SLC6A4 |
| CYP27B1 |
| MYEF2 |
| OXTR |
| NTRK1 |
| APOE |
| CD80 |
| SLC3A2 |
| HMOX1 |
| MMP3 |
| DDIT3 |
| CSF2 |
| NTRK2 |
| NTRK3 |
| GJA1 |
| GADD45A |
| MAT2A |
| ITGA5 |
| FAS |
| UBTF |
| MT2A |
| FN1 |
| DNAJA3 |
| JUN |
| IL1R1 |
| ACE |
| MMP19 |
| FGL2 |
| MAP3K12 |
| NFATC3 |
| STAT1 |
| SERPINE1 |
| SPI1 |
| GBA |
| EMILIN1 |
| GGT1 |
| MAP3K10 |
| DUSP8 |
| PRNP |
| RELB |
| CCND2 |
| TIMP2 |
| MDM2 |
| MTHFR |
| TNFRSF10A |
| ZNF253 |
| MAP3K13 |
| ATP2A2 |
| DLD |
| TAB1 |
| PPP4C |
| MAP3K4 |
| GSTP1DUSP4 |
| HOXA5 |
| HRH1 |
| PKD1 |
| ETS2 |
| NR2C2 |
| TFRC |
| TOM1 |
| ETS1 |
| TAB2 |
| MAP2K7 |
| POLD2 |
| DUSP10 |
| HMBS |
| CDK5 |
| GNRHR |
| AMD1 |
| NCL |
| APEX1 |
| MAT2A |
| MAP3K11 |
| MAPK1 |
| CHRM3 |
| NIN |
| MAPK9 |
| FASN |
| MAPK7 |
| HFESO |
| D1PD |
| HA1T |
| GFB1 |
| MAP2K5 |
| HMGA1 |
| ZNF480 |
| SOD2 |
| MAP4K1 |
| ATF2 |
| ZAKMETIER5 |
| CD79A |
| MAP2K4 |
| AKT1 |
| MTHFR |
| JAK3 |
| TCIRG1 |
| HLA-ASRR |
| HLA-CPEX3 |
| CDC42 |
| ITGAX |
| MAP3K1 |
| RB1 |
| MAP3K3 |
| APPTBXAS1 |
| PSAPERCC1 |
| MAPK3 |
| MAPK11 |
| CLUTR |
| AF6 |
| ME2 |
| MYBVDR |
| MAP3K5 |
| CYP1A2 |
| NFKB |
| 2IGFBP2 |
| MAP3K2 |
| MAP3K7 |
| NRAS |
| MAP3K11 |
| HBA1 |
| HLA-B |
| MAPK8 |
| GM2A |
| ABCA1 |
| LCP1 |
| ALDOA |
| PPARA |
| SMAD7 |
| SLC6A6 |
| CSTB |
| ICAM1 |
| LOX |
| TP53 |
| PLAUR |
| NXN |
| NAT1 |
| ITGA6 |
| HEXB |
| MAP3K9 |
| JAK3 |
| TCIRG1 |
| CHRM4 |
| HLA-DPB1 |
| PLA2G4A |
| OAS2 |
| PDE5A |
| HSPA1A |
| COL1A1 |
| TNC |
| GLRXS |
| HC1 |
| MGAT5B |
| PTGS1CHRM5 |
| TNFRSF8 |
| CYP11A1 |
| CD44 |
| GNRH1 |
| ACSL3 |
| MEP1B |
| CA2 |
| GAB1 |
| LENEP |
| MAPK8 |
